# Supplementary material for: Spondweni virus infection in pregnant rhesus macaques causes placental pathology without apparent fetal harm
Source: bioRxiv. 2026 Aug 10:2026.08.10.743875. Preprint. [Version 1] doi: 10.64898/2026.08.10.743875 (PMC13640649; doi:10.64898/2026.08.10.743875)
Supplement: Supplementary file 1 [file NIHPP2026.08.10.743875v1-supplement-1.pdf]

bioRxiv preprint doi: https://doi.org/10.64898/2026.08.10.743875; this version posted August 10, 2026. The copyright holder for this preprint (which was not certified by peer review) is the author/funder, who has granted bioRxiv a license to display the preprint in perpetuity. It is made available under a CC-BY 4.0 International license.

Supplemental Information

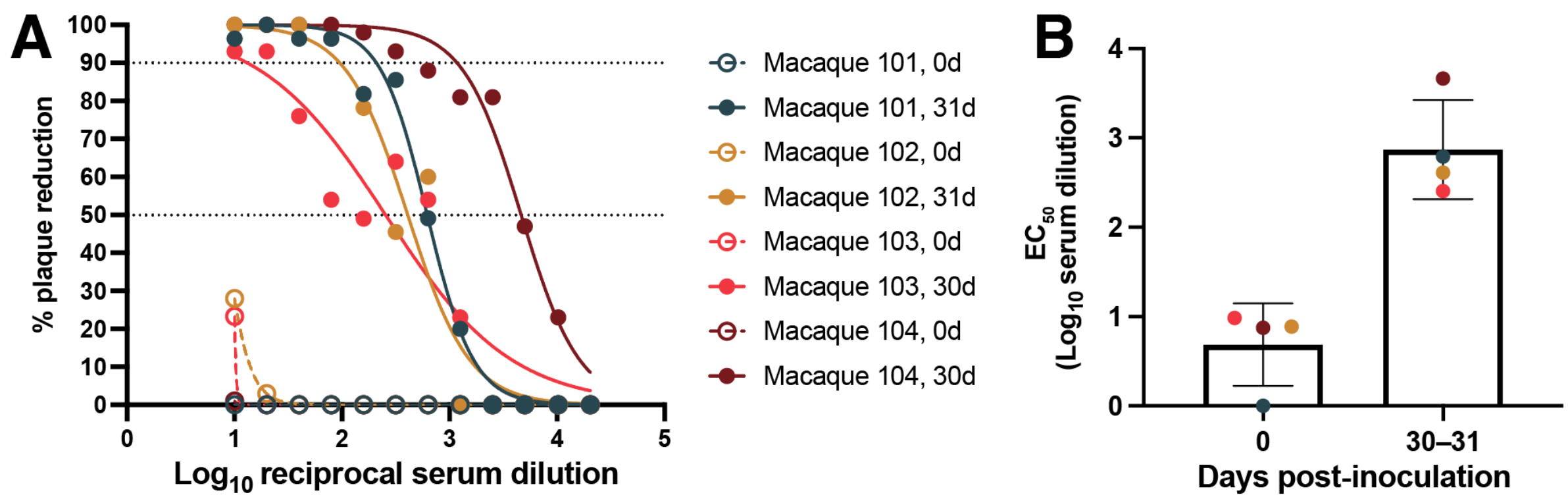

S1 Fig. Neutralizing antibody responses to Spondweni virus at 0 and 30–31 days post-inoculation. (A) Plaque-reduction neutralization curves for each macaque at day 0 (pre-inoculation) and 30–31 days post-inoculation (dpi). The y-axis shows percent plaque reduction, and the x-axis shows $\log_{10}$ reciprocal serum dilution. The horizontal grey lines indicate 90% and 50% plaque reduction. **(B)** EC50 values ($\log_{10}$ serum dilution achieving 50% plaque reduction) for the same animals at 0 and 30–31 dpi. Points represent individual macaques (n = 4), and bars show group mean ± SD.

bioRxiv preprint doi: https://doi.org/10.64898/2026.08.10.743875; this version posted August 10, 2026. The copyright holder for this preprint (which was not certified by peer review) is the author/funder, who has granted bioRxiv a license to display the preprint in perpetuity. It is made available under a CC-BY 4.0 International license.

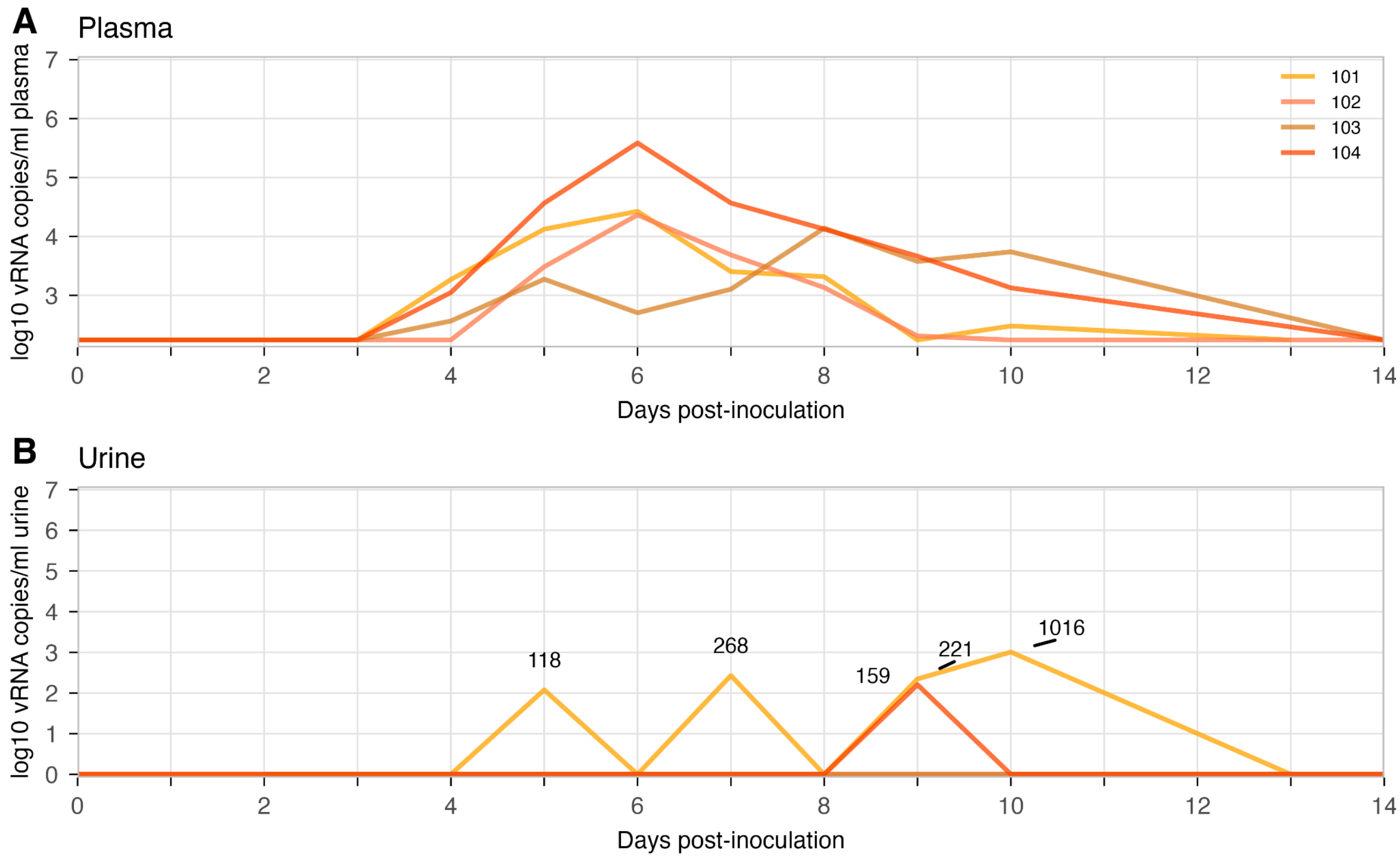

S2 Fig. Acute-phase plasma viremia and sporadic urinary detection in pregnant macaques inoculated with Spondweni virus (SPOV). (A) Longitudinal plasma viral RNA (vRNA) loads (log10 copies/mL) in SPOV-inoculated pregnant rhesus macaques during the first 14 days post-inoculation (dpi). Lines trace individual animals. The x-axis is truncated at 14 days post-inoculation to emphasize the acute phase of infection and does not include the two plasma samples that tested positive at days 23 and 31 (see **Fig 2**). **(B)** Urine vRNA loads for the same animals over dpi 0–14. All virus-positive urine samples are labeled with their corresponding viral load values. Values of zero represent samples that tested negative in two technical replicates. The assay lower limit of detection for SPOV in urine is 175 copies/mL, and values below it (e.g., 118 and 159 copies/mL) are considered detectable but not reliably quantifiable. Abbreviations: SPOV, Spondweni virus; vRNA, viral RNA; dpi, days post inoculation; LLOD, lower limit of detection.

bioRxiv preprint doi: https://doi.org/10.64898/2026.08.10.743875; this version posted August 10, 2026. The copyright holder for this preprint (which was not certified by peer review) is the author/funder, who has granted bioRxiv a license to display the preprint in perpetuity. It is made available under a CC-BY 4.0 International license.

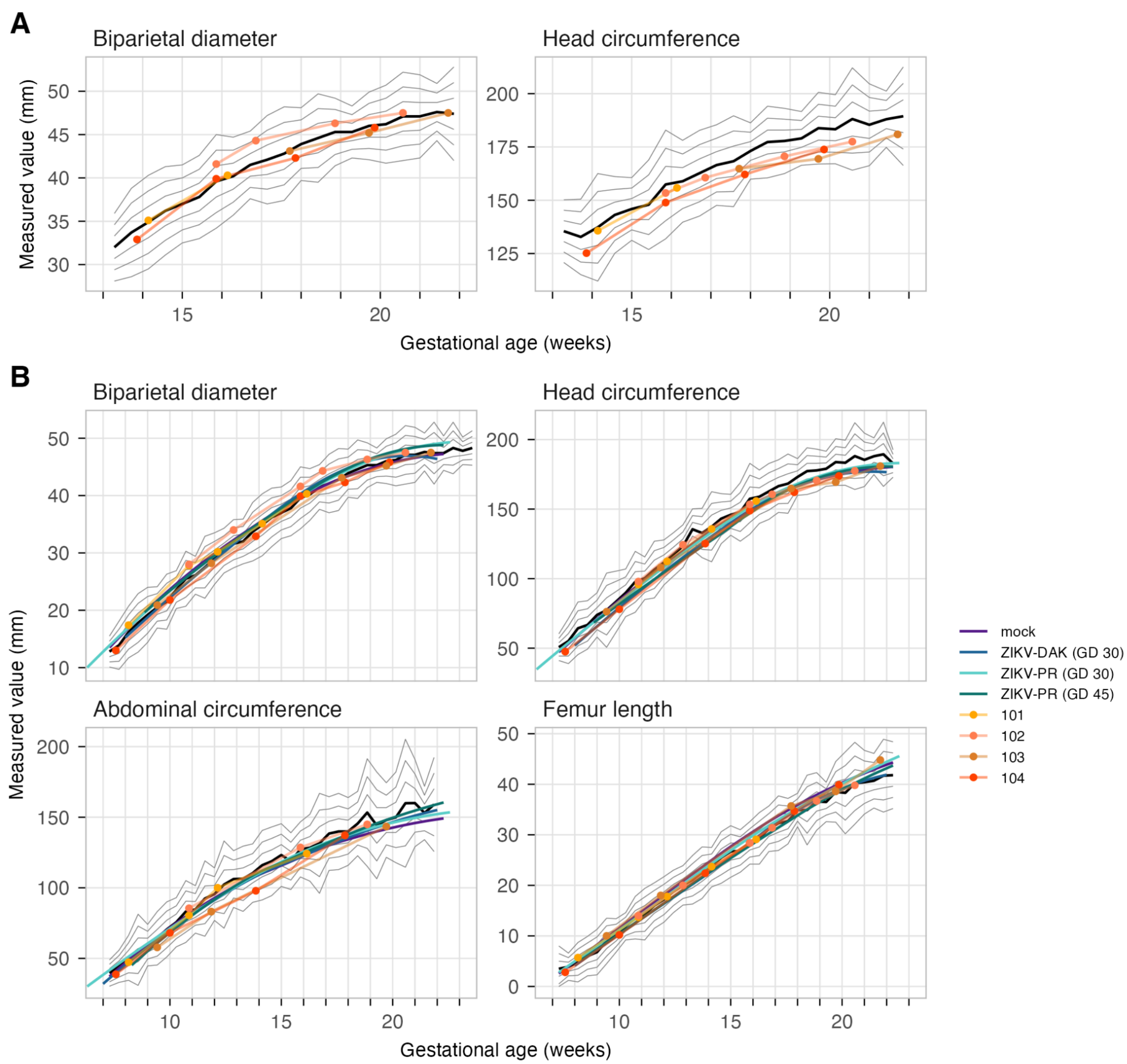

S3 Fig. SPOV-exposed macaques' fetal growth relative to normative curves and WNPRC cohorts. (A) Fetal growth trajectories for the SPOV-inoculated macaques' biparietal diameter and head circumference from 13–22 weeks of gestation. Each point (n = 4) represents one macaque, with lines connecting individual macaques through time. Normative fetal measurements are shown as a black line (mean) with flanking grey lines representing ±1, ±2, and ±3 standard deviations at each gestational age. **(B)** Full dataset of fetal biparietal diameter,

bioRxiv preprint doi: https://doi.org/10.64898/2026.08.10.743875; this version posted August 10, 2026. The copyright holder for this preprint (which was not certified by peer review) is the author/funder, who has granted bioRxiv a license to display the preprint in perpetuity. It is made available under a CC-BY 4.0 International license.

head circumference, abdominal circumference, and femur length. Fetal growth trajectory regressions are shown for mock-inoculated (purple), ZIKV-PR-inoculated (light blue), and ZIKV-DAK-inoculated (dark blue) macaques from prior studies [42].

bioRxiv preprint doi: https://doi.org/10.64898/2026.08.10.743875; this version posted August 10, 2026. The copyright holder for this preprint (which was not certified by peer review) is the author/funder, who has granted bioRxiv a license to display the preprint in perpetuity. It is made available under a CC-BY 4.0 International license.

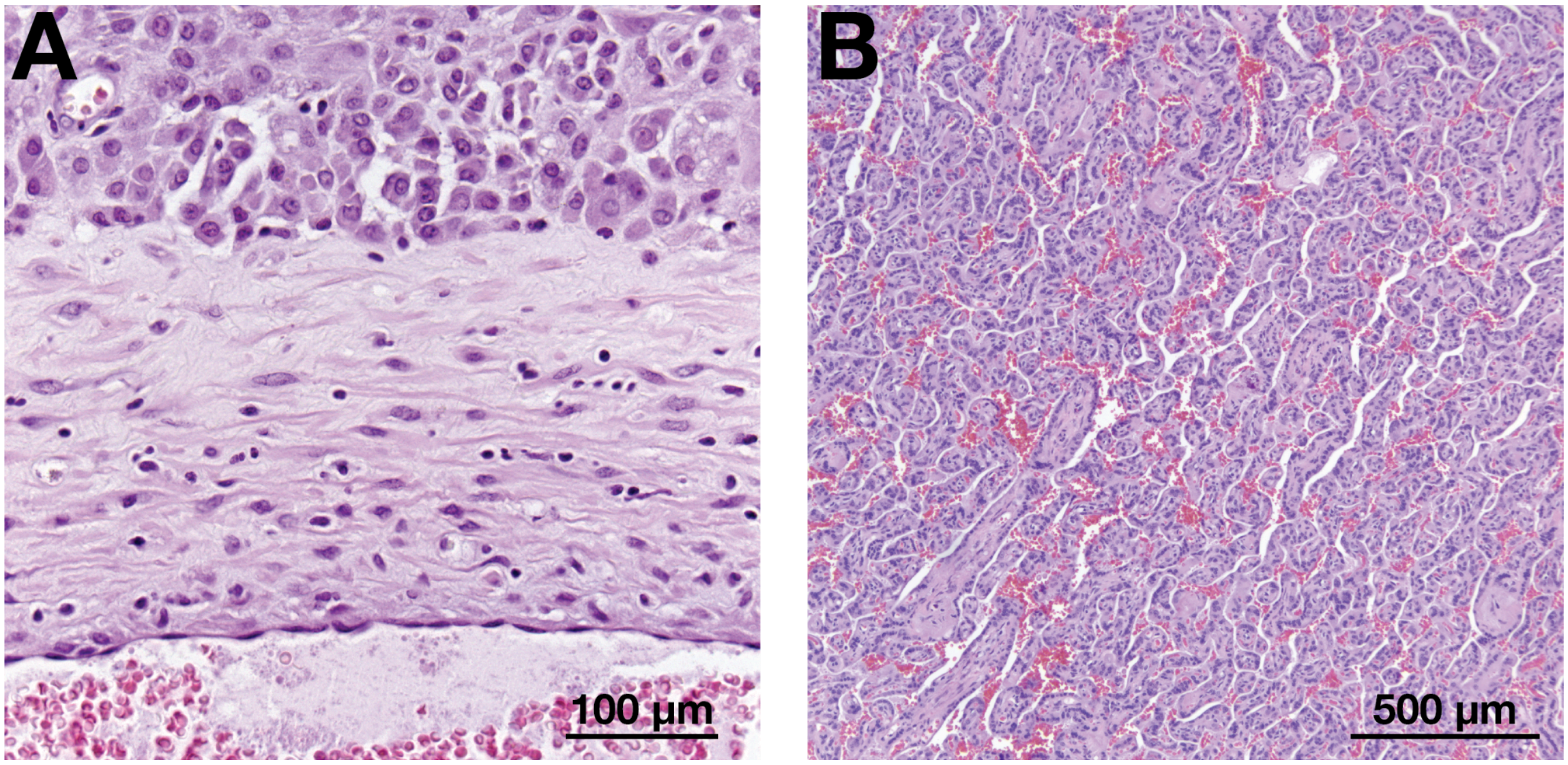

S4 Fig. H&E-stained normal basal-plate artery and chorionic villous parenchyma from rhesus macaque placentas. (A) Maternal side of the placenta (basal plate) at 40× showing a normal spiral artery with a wide lumen and a thin vessel wall. No inflammation of the vessel wall (vasculitis) is present. Scale bar = 100 µm. **(B)** Chorionic villous parenchyma at 10× with preserved branching villi and open spaces between villi (intervillous spaces) containing red blood cells. No villitis, thrombi, perivillous fibrin, or infarction is evident. Scale bar = 500 µm. Panel A serves as a normal reference for Fig 5; Panel B serves as a normal reference for Fig 6. Abbreviations: H&E, hematoxylin and eosin.

bioRxiv preprint doi: https://doi.org/10.64898/2026.08.10.743875; this version posted August 10, 2026. The copyright holder for this preprint (which was not certified by peer review) is the author/funder, who has granted bioRxiv a license to display the preprint in perpetuity. It is made available under a CC-BY 4.0 International license.

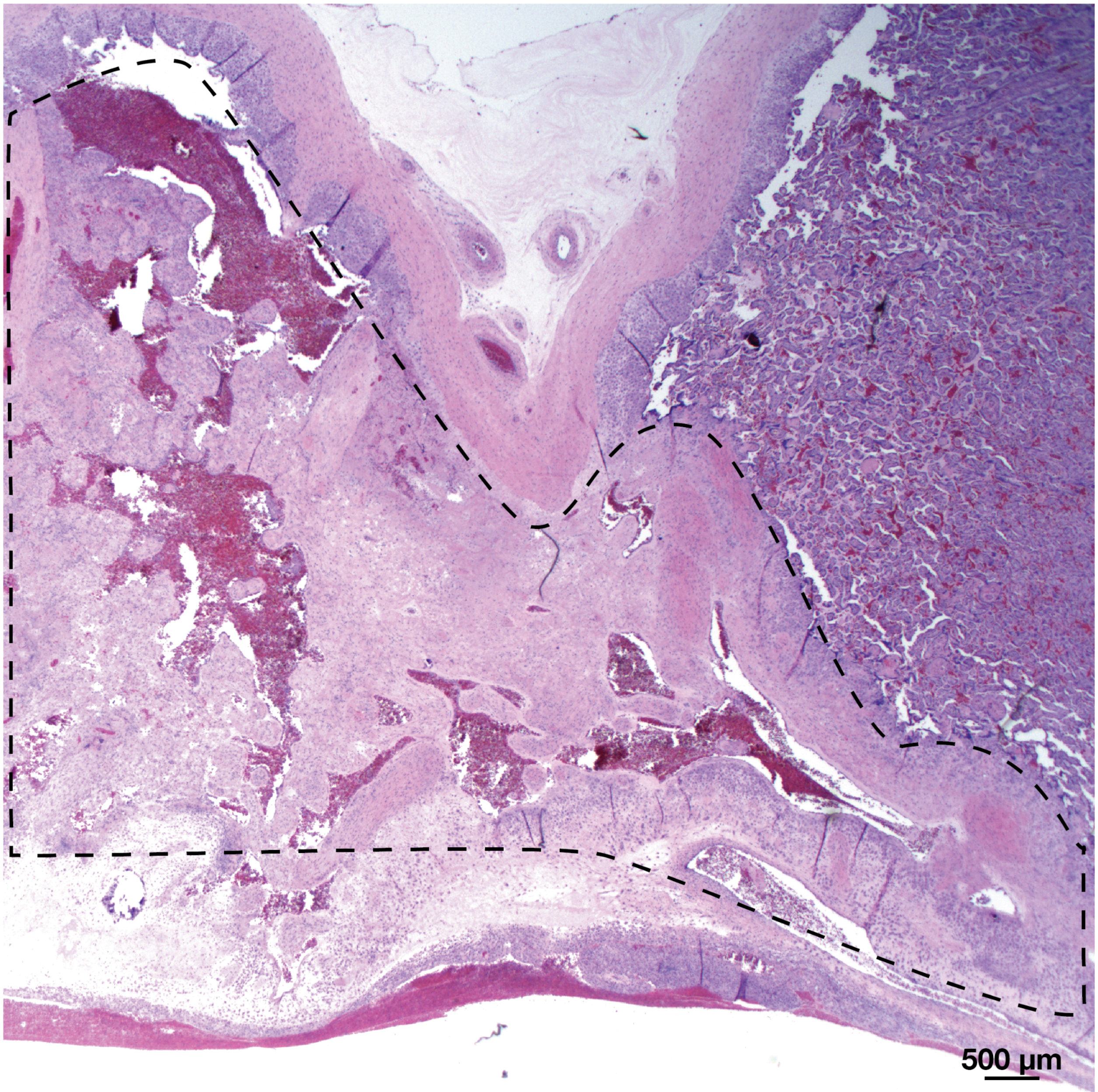

S5 Fig. Representative image of transmural ischemia of the placenta cross-section from macaque 104. H&E-stained full-thickness placental section at delivery from macaque 104. The dashed outline marks ischemic placental parenchyma extending from the basal plate (bottom) to the chorionic plate (top), consistent with transmural placental ischemia. The chorionic plate has fallen into a “V shape” due to necrosis and loss of supporting villous tissue. Adjacent uninvolved chorionic villi are visible at right for comparison.

bioRxiv preprint doi: https://doi.org/10.64898/2026.08.10.743875; this version posted August 10, 2026. The copyright holder for this preprint (which was not certified by peer review) is the author/funder, who has granted bioRxiv a license to display the preprint in perpetuity. It is made available under a CC-BY 4.0 International license.

Supplemental Table 1. List of fetal, maternal, and maternal-fetal tissues collected during fetal necropsy near term.

Fetal Tissues	
CNS	Terminal CSF collection
	Dura Mater
	Cervical spinal cord
	Thoracic spinal cord
	Lumbar spinal cord
	Cerebrum - right (10 sections)
	Cerebrum - left
	Cerebellum - 1 right-most median section
	Cerebellum - left
	Cerebellum - 3 right-most lateral section
	Cerebellum - 2 right
Ocular	Aqueous humor - right aspirate and aliquot
	Optic nerve - right
	Eye - left
	Sclera - right
	Cornea - right
	Retina - right
Cardiopulmonary	Pericardium
	Heart full thickness section
	Aorta - thoracic
	Lung
Reproductive	Sem vesicle & prostate/uterus
	Testis/Ovary
Musculoskeletal	Adipose tissue - omentum
	Epidermis/dermis of abdomen
	Muscle - quadriceps
Immune	Bone marrow - if possible R femur only
	Tonsil oropharyngeal LN
	Spleen
	Thymus
	Submandibular LN
	Tracheobroncial LN
	Mesenteric LN
	Axillary LN
	Inguinal LN
Gastrointestinal	Esophagus
	Stomach
	Duodenum
	Jejunum
	Ileum
	Cecum
	Colon
	Liver
	Tongue
Urinary	Urinary bladder
	Kidney - right and left
	Urine - aspirate
Endocrine	Thyroid
	Adrenal Gland
	Pancreas

Maternal tissues
Spleen
Liver
Mesenteric LN
Uterus - Placental bed (point of attachment)

Maternal-fetal interface
Amniotic fluid
Umbilical cord blood
Chorionic plate
Fetal membranes (taken distant from placenta)
Placenta disc 2 full tickness
Placental disc 1 periphery - no decidua
Placental disc 2 periphery - no decidua
Decidua - removed from placenta (Basalis)
Inter-placental collateral vessels
Umbilical cord

bioRxiv preprint doi: https://doi.org/10.64898/2026.08.10.743875; this version posted August 10, 2026. The copyright holder for this preprint (which was not certified by peer review) is the author/funder, who has granted bioRxiv a license to display the preprint in perpetuity. It is made available under a CC-BY 4.0 International license.

Supplemental Table 2. Placental histopathology by animal. H&E-stained placental sections collected at delivery were reviewed for two discs per pregnancy (center cut plus cotyledon samples). Cells list the dominant findings across both discs for each animal. Abbreviations: MVM = maternal vascular malperfusion and TM = transmural.

Animal	MVM (spiral arteries)	Vasculitis	Ischemia / Necrosis	Villitis	Thrombosis / hemorrhage / mineralization
101	Minimal/rare persistently muscularized decidual arteris and minimal acute deciduitis &endometritis.	Not observed.	**Present**. Small TM foci of ischemia (7% and 6% of total parenchyma of the two discs respectively). Multifocal non-transmural ischemic foci	**Present**. Focal chronic villitis (subjacent to basal and chorionic plates).	**Present**. Multifocal thrombosis (~50–60% of vessels) with moderate intravascular fibrin; mild multifocal mineralization.
102	**Present.** Moderate multifocal persistently muscularized decidual arteries. (29% overall).	**Present**. Lymphoplasmacytic vasculitis of large placental vessels.	**Present**. Large TM focus of ischemia near the umbilical insertion (~66% of disc 1 parenchyma) and broad TM regions of ischemia in disc 2. Extensive multifocal non-transmural parenchymal ischemia/necrosis with lateral ischemia.	**Present**. Mild multifocal chronic lymphohistiocytic villitis.	**Present**. Mild perivillous fibrin; chronic intervillous hemorrhage; moderate multifocal mineralization; chronic retroplacental hemorrhage with hemosiderosis.
103	**Present**. Mild multifocal persistently muscularized decidual arteries (~9% overall).	Not observed.	**Present**. Large TM foci of ischemia measuring 28% and 26% of total parenchyma of the two discs. Moderate to marked multifocal non-transmural ischemia with hemorrhage	**Present**. Minimal-to-mild acute and chronic focal villitis adjacent to ischemic regions.	**Present**. Mild multifocal mineralization; hemorrhage in decidua basalis; 80% thrombosed on primary disc and 40% thrombosis with lateral retroplacental hemorrhage on the secondary disc.
104	**Present**. Mild multifocal persistently muscularized decidual arteries and mild chronic deciduitis (~8% overall) in one disc and 0% in the other disc.	Not observed.	**Present**. One small TM focus of ischemia (0.05% of disc 1 parenchyma) and a single TM focus of ischemia (18%of disc 2 parenchyma). Multifocal non-transmural ischemia with organizing fibrin & hemorrhage	**Present**. Mild multifocal acute and chronic villitis.	**Present**. Focal intervillous hemorrhage; ~20–30% thrombosed across discs; minimal villous mineralization; minimal perivillous fibrin.
